# The prevalence of sarcopenia in Parkinson’s disease and related disorders- a systematic review

**DOI:** 10.1007/s10072-023-07007-0

**Published:** 2023-08-18

**Authors:** Ashley Hart, Laura Cordova-Rivera, Fred Barker, Avan A. Sayer, Antoneta Granic, Alison J. Yarnall

**Affiliations:** 1 Brain and Movement Research Group, Campus for Ageing and Vitality, Translational and Clinical Research Institute, Newcastle University, Newcastle Upon Tyne, UK; 2 AGE Research Group, NIHR Newcastle Biomedical Research Centre, Newcastle University, Newcastle Upon Tyne, UK; 3 Newcastle Upon Tyne Hospitals NHS Foundation Trust, Newcastle, UK; 4 School of Health and Life Sciences, Centre for Rehabilitation, Teesside University, Middlesbrough, UK; 5 Northumbria Healthcare NHS Foundation Trust, Newcastle upon Tyne, UK

**Keywords:** Sarcopenia, Parkinson’s disease, Parkinsonian disorders, Associations

## Abstract

**Background:**

The prevalence of sarcopenia (reduced skeletal muscle strength and mass), Parkinson’s disease (PD) and Parkinson’s related disorders (PRD) all increase with age. They also share risk factors and pathogenetic features. An increased prevalence of sarcopenia in PD and PRD than the general population was thus postulated.

**Methods:**

Four databases were searched using predefined literature search strategies. Studies conducted in participants with PD or PRD reporting the prevalence of sarcopenia and those providing data to compute the prevalence were included. Pre-sarcopenia, probable/possible sarcopenia and confirmed sarcopenia were defined according to the main sarcopenia working groups. Risk of bias was assessed using the AXIS tool.

**Results:**

1978 studies were identified; 97 assessed in full; 14 met inclusion criteria. The median study quality score was 15/20. The range of probable sarcopenia was 23.9 to 66.7%, and it did not change after excluding PRD participants. The prevalence of confirmed sarcopenia in participants with any parkinsonian disorder ranged from 2 to 31.4%. Including just PD participants, the range was 10.9 to 31.4%. In studies with controls, sarcopenia was more prevalent in PD and PRD. There was a positive non-significant trend between severity of motor symptoms and prevalence of sarcopenia or components of sarcopenia. High heterogeneity precluded meta-analysis, therefore there was insufficient evidence to conclude whether sarcopenia is more prevalent in PD or PRD.

**Conclusions:**

Probable and confirmed sarcopenia are common in PD and PRD and they may be associated with disease severity. This co-occurrence supports the value of screening for sarcopenia in parkinsonian populations.

**Supplementary Information:**

The online version contains supplementary material available at 10.1007/s10072-023-07007-0.

## Introduction

### Background

Parkinson’s disease (PD) is the fastest growing neurological condition globally [1]. As the population ages, the burden of PD and the inherent demands on health and social care systems are set to grow. Parkinson’s related disorders (PRD) such as multiple system atrophy (MSA), progressive supranuclear palsy (PSP), and corticobasal syndrome (CBS) also have negative implications in later life [2]. Thus, it is imperative that we find strategies for optimising health in those with PD and PRD over their life course.

Sarcopenia is the loss of skeletal muscle strength and mass that occurs commonly with ageing [3]. It is associated with falls, impaired ability to perform activities of daily living [4], loss of independence and poorer quality of life in older people. There is evidence that resistance exercise is beneficial in sarcopenia [5], and that it is well accepted as a treatment by patients [6], yet sarcopenia remains underdiagnosed [7]. Developing a standardised approach to the recognition and management of sarcopenia is a core aim of working groups such as the European Working Group in Sarcopenia in Older People (EWGSOP, EWGSOP2) [4, 8], the Asian Working Group for Sarcopenia (AWGS) [9] and the International Working Group for Sarcopenia (IWGS) [10]. However, the recommended definitions and measurement cut offs vary between these groups.

There is evidence to suggest sarcopenia occurs more frequently in PD than would be expected in age matched cohorts [11–13] Furthermore, in prodromal PD the prevalence of components of sarcopenia (i.e., lower values of muscle mass, strength and/or physical performance) has been correlated with the Movement Disorder Society Unified Parkinson’s Disease Rating Scale (MDS-UPDRS) part III score [14], a measure of motor impairment [15], indicating a possible association between disease stage and presence of sarcopenia. The overlap between the two conditions may be caused by reduced activity levels in PD [16]. Additionally, reduced caloric intake could lead to a reduction in muscle mass and quality. Symptoms of depression and anorexia in addition to dopaminergic medication side effects such as nausea may also contribute [17].

Shared pathogenetic features have also been noted between sarcopenia and PD. Parkinsonism arises from disruption to the basal ganglia, a group of subcortical nuclei whose primary function is to initiate movement [18]. In PD this disruption may be caused by mitochondrial dysfunction, impairment of protein clearance, neuroinflammation and oxidative stress [19]. These processes ultimately lead to aggregated alpha synuclein forming Lewy bodies which deposit throughout the nervous system [20]. MSA pathology is also underpinned by alpha synuclein, whereas PSP and CBS are caused by aberrant tau protein [21]. Shared features of PD with sarcopenia include neuroinflammation mediated by interleukin 6 (IL6). IL6 has been linked to loss of muscle mass and poor physical performance in both older adults and PD [14] and a reduction in the number of motoneurons has been observed both in sarcopenia and PD [17]. In addition, mitochondrial dysfunction has been demonstrated in non-neuronal tissues of people with PD [22] and it has been shown that mitochondrial abnormalities are more frequent in sarcopenic muscles compared to healthy aged muscle [23].

Despite this putative overlap, estimates of the prevalence of sarcopenia in PD vary widely and research into sarcopenia in PRD is sparse. To date, only one systematic review and meta-analysis has investigated the prevalence of sarcopenia in PD with no analysis of PRD [24]. Compared with this study by Cai et al., the current systematic review: (a) encompasses considerable further and more updated research; (b) applies more stringent inclusion criteria (i.e. exclusively including studies that correctly use recognised guidance for sarcopenia or probable sarcopenia diagnosis from one of the three main working groups); (c) uses more rigorous methods of analyses by following guidance on the appropriateness of any potential meta-analysis [25]; (d) examines the relationship between PD disease severity and sarcopenia; and (e) compares the sarcopenia prevalence rates in PD and PRD with those reported in the general population.

### Review aims

We therefore aimed to systematically review:

1. the prevalence of sarcopenia in populations with PD and PRD, as per definitions proposed by relevant sarcopenia working groups
2. if the prevalence of sarcopenia varies with motor severity
3. if the prevalence of sarcopenia differs between those with PD and other PRD
4. how the prevalence rates of sarcopenia in PD and PRD compare to the prevalence rates in the general population.

## Methods

This review was performed in accordance with the Preferred Reporting Items for Systematic Review and Meta-Analyses (PRISMA) guidelines [26], and was registered on the International Prospective Register of Systematic Reviews (PROSPERO; http://www.crd.york.ac.uk/prospero) on 16/09/2020 as CRD209477.

### Search strategy

We developed literature search strategies using medical subject headings and text words related to sarcopenia and parkinsonism (Table 1). MEDLINE (Ovid interface), EMBASE (OVID interface), Scopus and Web of Science were searched. To ensure literature saturation we also scanned reference lists of studies identified through the search.

**Table 1:** Search strategy

Search strategy: [(#1) AND (#2)] NOT (#3)	
#1	(exp sarcopenia/) OR (muscle weakness/ or muscular atrophy/) OR (muscle wasting.mp.) OR (muscle mass.mp.) OR (grip strength.mp)
#2	(parkinsonian disorders/ or multiple system atrophy/ or parkinson disease/ or supranuclear palsy, progressive/) OR [(corticobasal degeneration or cortico-basal degeneration).mp.] OR [(corticobasal syndrome or cortico-basal syndrome).mp.]
#3	rat* OR mouse OR mice OR animal*.mp

Searches were run from database inception date to 10^th^ October 2022. No restrictions were placed on study location or language.

### Inclusion criteria

Cross-sectional, randomised-control trials (baseline measurements), case control or cohort studies conducted in patients with PD or PRD (together referred to as parkinsonian participants) reporting on the prevalence of sarcopenia or those that provide enough data to compute this using EWGSOP [8], EWGSOP2 [4], AWGS [9] or IWGS [10] guidance were included. At inception of this review, we had intended to include studies on participants with MSA, PSP and CBS. The search yielded studies which additionally included participants with vascular parkinsonism (VaP) and Lewy body dementia (LBD).

Intervention studies from which we could extract data on the prevalence of sarcopenia in PD or PRD at baseline were included. For longitudinal studies commenting on the change in prevalence over time, the latest time point in the study was used.

### Exclusion criteria

Editorials, letters, case reports and conference abstracts were excluded in addition to studies conducted in animals. Studies conducted on nursing home residents were excluded to avoid the prevalence of sarcopenia being confounded by the presence of frailty.

### Study selection

Using Endnote software, we screened for duplicate studies and excluded them. Three of the review authors (LCR, AH, FB) independently screened titles and abstracts, obtaining full reports for all that appeared to meet inclusion criteria and those with any uncertainty. Any disagreements were resolved through discussion and reasons for excluding trials recorded. Where consensus could not be reached, an additional reviewer was consulted (AJY).

We contacted authors of studies which estimated the prevalence of sarcopenia in a cohort that included a subgroup of parkinsonian participants to obtain relevant data. We also contacted authors reporting on sarcopenia data from which pre-sarcopenia, probable/possible sarcopenia, or confirmed sarcopenia could have been estimated according to the sarcopenia working groups referenced. A maximum of two contact attempts was applied to all authors.

### Quality assessment and risk of bias

Two researchers (AH, FB) independently assessed the studies using the AXIS tool [27]. Any longitudinal or intervention studies were assessed using this tool intended for use on cross-sectional studies as our outcomes of interest were cross-sectional in nature. Any disagreements that could not be resolved through discussion were resolved by a third reviewer (AJY).

### Data collection

Data were extracted by three reviewers (LCR, AH, FB) independently using a standardised proforma. Extracted data included author, year, country, type of study and sample size; average age of participants; type of parkinsonian disorder; measurement of sarcopenia used; details of controls; disease severity; outcome measures. Outcome measures included any measure used to define sarcopenia in the EWGSOP [8], EWGSOP2 [4], AWGS [9] or IWGS [10] guidance (Table 2 and Supplementary Table-1).

**Table 2:** Sarcopenia definitions according to sarcopenia working groups

EWGSOP	Pre-sarcopenia = low muscle mass, sarcopenia = low mass + function (strength or performance), severe sarcopenia = low mass, strength and physical performance
EWGSOP2	Probable sarcopenia = low muscle strength, confirmed sarcopenia = low muscle strength + low muscle mass or quality, severe sarcopenia = low muscle strength + low muscle mass/quality + reduced physical performance
AWGS	Possible sarcopenia = either low muscle strength or low physical performance only, sarcopenia = loss of muscle mass + low muscle strength + /or low physical performance
IWGS	Sarcopenia = gait speed of less than 1 m/s and an objectively measured low muscle mass

*EWGSOP* European Working Group on Sarcopenia in Older People (2010), *EWGSOP2* updated EWGSOP guideline (2019), *AWGS* Asian Working Group for Sarcopenia, *IWGS* International Working Group for Sarcopenia

Pre-sarcopenia was defined according to EWGSOP as low muscle mass.

Probable and possible sarcopenia were defined in accordance with EWGSOP2 (as low muscle strength) or AWGS (low muscle strength or physical performance), respectively.

The EWGSOP defines confirmed sarcopenia as low skeletal muscle mass and function (strength or performance); EWGSOP2 as low muscle strength and low muscle mass or quality; AWGS as loss of skeletal muscle mass and low muscle strength or low physical performance; and IWGS as gait speed of less than one meter per second and an objectively measured low muscle mass.

Within sarcopenia definitions, skeletal muscle strength was measured by grip strength, knee flexion/extension or time to stand from a chair five times. Skeletal muscle mass was assessed using dual Xray absorptiometry (DXA) or bioelectrical impedance analysis (BIA) (estimating body composition through impedance of an electrical current). Physical performance was evaluated using the timed up and go (TUG) test (time taken to stand from a seat, walk 3 m, turn around, walk back to chair and sit down), short physical performance battery (SPPB) (ability to hold posture with feet side by side, semi-tandem and in tandem); or measures of gait speed across four or six meters (4mGS, 6mGS) (Supplementary Table-1).

Disease severity was measured using the MDS-UPDRS part III [15], where higher scores are associated with higher PD-related impairments.

### Data synthesis

Analyses were conducted using SPSS 26 and RevMan 5. Plot Digitazer online software (https://plotdigitizer.com/) was used to extract data from figures, when relevant [28]. The range of prevalence of sarcopenia in parkinsonian disorders were presented. For studies which provided data for control groups, odds ratios were calculated and presented on a forest plot. Studies which met inclusion criteria were assessed for clinical and methodological heterogeneity. Statistical heterogeneity was then assessed using the I^2^ statistic with a random effects model. It was agreed that in the case of finding high clinical, methodological or statistical heterogeneity in the identified literature, meta-analyses would not be performed [25].

Due to the small number of studies which assess sarcopenia in PRD, these are presented as a narrative summary.

Finally, we compared the prevalence of sarcopenia and probable sarcopenia in the PD and PRD participants with that of the general population. Figures for the general population were taken from the most up to date and comprehensive studies found from a literature search [29, 30].

## Results

### Search results and description of studies

**Fig. 1:**
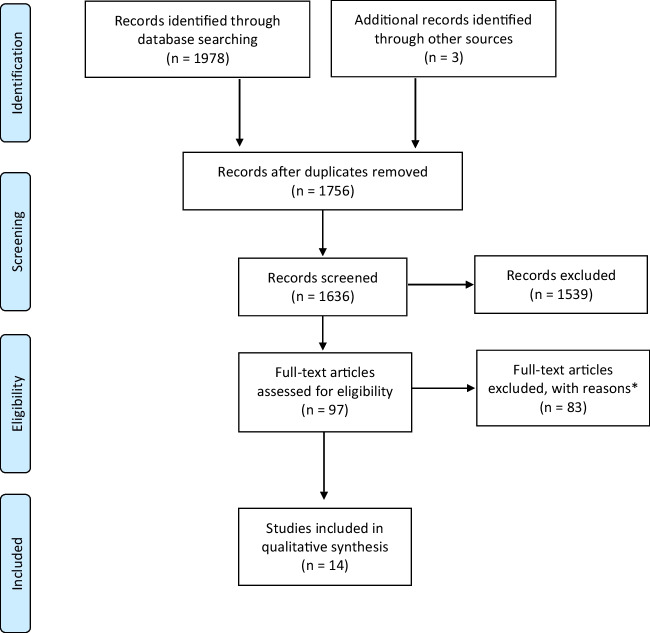
PRISMA Flow Diagram Literature search. Updated October 10th, 2022. Summary of records identified. * Reasons may include articles not included due to authors not providing relevant data after second request

We identified 1981 records up to the 10^th^ of October 2022, including three record identified by hand searching. After removal of duplicates (*n* = 225) and non-relevant study designs, 1539 articles were excluded post screening of titles and abstracts leaving 97 studies to be assessed in full. Among the 16 articles identified [12, 13, 17, 31–43], one article [42] was excluded to avoid data duplication after the authors confirmed that this cohort was a sub-analysis of an already identified article [33]. A second article [43] was also excluded as its population was taken from a nursing home, as per exclusion criteria. As such, 14 articles were found to meet inclusion criteria and were included in the analyses and narrative summary of this review [12, 13, 17, 31–41]. Figure 1 presents the PRISMA Flowchart of the summary of literature identified in the searches.

From the fourteen articles included in the synthesis [12, 13, 17, 31–41], ten [12, 13, 31–38] gave estimates of the prevalence of confirmed sarcopenia, including seven [12, 13, 31, 32, 35, 37, 38] that additionally gave estimates of either probable sarcopenia (EWGSOP2) [12, 37] or low hand grip strength (dynapenia, EWGSOP cut off) as component of sarcopenia [13, 31, 32, 35, 38]. Three articles [17, 39, 40] gave estimates of the prevalence of probable sarcopenia or low grip strength only. One study [41] was included following contact with authors who provided the raw data for hand grip strength and bioimpedance analysis, from which we were able to compute an estimate of both probable and confirmed sarcopenia using the EWGSOP2 criteria.

The mean age of parkinsonian participants across studies was 69.8 (range 65.4–79.9) years and 59.0% were male. The mean age in studies which only included idiopathic PD participants was 69.7 (range 65.4–79.9) years and 59.4% were male.

Of the fourteen included studies, seven [13, 31, 32, 34, 35, 38, 39] used the original EWGSOP criteria [8], five [12, 17, 33, 40, 41] used the revised EWGSOP2 criteria [4] and one [36] used AWGS criteria [9]. One study [37] used the EWGSOP2 algorithm with AWGS cut offs, and another [38] gave an estimate of the prevalence of sarcopenia using both EWGSOP and IWGS. Therefore, to avoid duplication of these data in our analyses, we included data from EWGSOP only. This was chosen over IWGS as no other studies had used IWGS criteria thus including this would have increased overall heterogeneity in the analyses.

Five [13, 34–37] of the studies included a control population. There was a high degree of clinical heterogeneity between control groups across studies. Three studies [31, 32, 34] reported information on PRD. Eight studies [12, 31–33, 36, 37, 39, 40] reported the average MDS-UPDRS III score of PD participants. However, since only three of these articles [12, 32, 33] reported MDS-UPDRS III scores by confirmed sarcopenia status, further statistical analysis was ruled out. Two studies reported MDS-UPDRS III by probable sarcopenia or low handgrip strength [32, 40].

### Study quality

Of the fourteen studies included, one was a randomised control trial and one was a cohort study; the remainder were cross-sectional studies. The median AXIS score was 15/20 (range 11–18) with a higher score indicating better quality (Supplementary Table-2).

### Prevalence of sarcopenia

Table 3 shows the baseline characteristics of the studies included, and Table 4 shows the prevalence of sarcopenia and probable sarcopenia based on three sarcopenia algorithms and cut-offs for low muscle strength (using grip strength), mass (from BIA or DXA) and physical performance (from gait speed and TUG). In the included studies, the range of the prevalence of confirmed sarcopenia in participants with any parkinsonian disorder was 2–31.4%. Including just studies on PD participants the range was 10.9–31.4%. The range of sarcopenia prevalence according to the different guidelines was 2–31.4% for EWGSOP and 10.9–22.2% for EWGSOP2. The only study [36] that used AWGS guidance estimated a prevalence of sarcopenia in PD of 17.2%. As mentioned previously, one study [38] used both EWGSOP and IWGS guidance, with only the former guidance being reported in this review.

**Table 3:** Demographic characteristics of included studies

Reference	Year	Country	n (PD/PRD)	Mean age	% Male	Disease type	Control
Yarnall et al. [12]	2020	UK	9	79.9	77.8	PD	None
Yazar et al. [13]	2018	Turkey	166	71.6	50.0	PD	Hospital staff
Lima et al. [17]	2020	Brazil	218	67.9	57.3	PD	None
Barichella et al. [31]	2019	Italy	150	67.7	64.00	PD, MSA, PSP, VaP	None
Barichella et al. [32]	2016	Italy	364	72.8	53.3	PD, VaP, PSP, MSA, LBD, CBD, other	None
da Luz et al. [33]	2020	Brazil	77	65.4	58.4	PD	None
Krenovsky et al. [34]	2020	Germany	74	70.9	54.1	PD, LBD, PSP, CBD, MSA	Age and sex matched
Ozer et al. [35]	2019	Turkey	70	68.3	58.6	PD	Outpatient geriatrics
Tan et al. [36]	2018	Malaysia	93	66.0	54.8	PD	Spousal/sibling
Tan et al. [37]	2020	Malaysia	73	68.2	55.9	PD	Spousal/sibling
Vetrano et al. [38]	2018	Italy/Sweden	210	73.0	61.9	PD	None
Lindskov et al. [39]	2016	Sweden	65	68.1	53.9	PD	None
Roberts et al. [40]	2015	UK	57	71.8	59.7	PD	None
Bernhard et al. [41]	2018	Germany	46	67.6	70.0	PD	None

*PD* Parkinson’s disease, *MSA* multiple system atrophy, *PSP* progressive supranuclear palsy, *CBD* corticobasal degeneration, *VaP* vascular parkinsonism, *LBD* Lewy body disease

**Table 4:** Prevalence of sarcopenia and outcome measures

		Outcomes					
References	Guideline used	Sarcopenia % PD and PRD	Sarcopenia % Control	Low HGS %	Low muscle mass %	Other	Mean MDS-UPDRS III^
Yarnall et al. [12]	EWGSOP2	22.2	-	66.7	55.6 SMMI	4mGS < 0.8 m/s; 55.6%	25.1
Yazar et al. [13]	EWGSOP	25.9	12.4	71.0	-	-	-
Lima et al. [17]	EWGSOP2	-	-	47.4	-	-	-
Barichella et al. [31]	EWGSOP	2.0	-	52.0	2.0 SMMI	4mGS < 0.8 m/s; 26.7%	22.9
Barichella et al. [32]	EWGSOP	6.6	-	75.5	7.4 SMMI	4mGS < 0.8 m/s; 61.3%	26.3
da Luz et al. [33]	EWGSOP2	19.5	-	-	-	-	25.3
Krenovsky et al. [34]	EWGSOP	13.5	0	-	-	-	-
Ozer et al. [35]	EWGSOP	31.4	17.6	50.0^$^	-	-	-
Tan et al. [36]	AWGS	17.2	10.3	-	20.4 SMMI	-	32.9
Tan et al. [37]	EWGSOP AWGS cut off	26.0	4.3	56.2	31.5 SMMI	-	36.1
Vetrano et al. [38]	EWGSOP	24.2	-	76.7	43.3 ASMM	4mGS < 0.8 m/s; 51.0%	-
Lindskov et al. [39]	EWGSOP	-	-	26.2	-	-	14.0
Roberts et al. [40]	EWGSOP2	-	-	28.1^+^	-	-	23.3
Bernhard et al. [41]	EWGSOP2	10.9	-	23.9	23.9 ASMM	TUG ≥ 20 s; 15.2%	-

*SMMI* Skeletal muscle mass index, *ASMM* Appendicular skeletal muscle mass, *HGS* hand-grip strength, *4mGS* 4-m gait speed test, *TUG* Timed up and go Test, *MDS-UPDRS* Movement Disorder Society Unified Parkinson’s Disease Rating Scale. *EWGSOP* European Working Group for Sarcopenia in Older People, *AWGS* Asian Working Group for Sarcopenia *Median reported. ^ Scores reported correspond to the whole PD/PDR cohort. ^$^HGS cut off used different from those proposed by EWGSOP. ^+^ Data calculated from figure using PlotDigitised.com App

The range of probable sarcopenia (defined by the EWGSOP2 criteria as low muscle strength [< 27 kg for men, < 16 kg for women]) was 23.9–66.7% in participants with any parkinsonian disorder. The range of prevalence of low hand grip strength (i.e., dynapenia) as per EWGSOP (< 30 kg for men < 20 kg for women) ranged between 26.2–76.7%. Studies including participants with PRD were included in the latter prevalence range. These values did not change when excluding these studies.

**Fig. 2:**
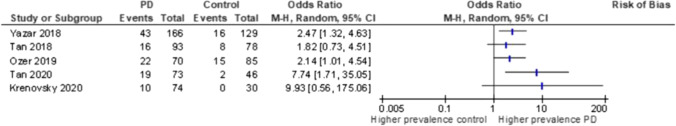
Forest plot showing odds ratios of studies which included prevalence of sarcopenia in those with parkinsonian disorders and controls. Yazar 2018 [13], Krenovsky 2020 [34], Ozer 2019 [35], Tan 2018 [36], Tan 2020 [37]

Figure 2 demonstrates the odds ratios of prevalence of sarcopenia in those with PD or parkinsonian disorders compared to controls. All studies [13, 34–37] reported a trend towards greater odds ratios indicating a higher prevalence of sarcopenia in PD compared with controls participants, with three [13, 35, 37] out of five studies reaching statistical significance. Meta-analysis was precluded by several examples of clinical heterogeneity. For instance, three studies using EWGSOP criteria [13, 34, 35], one [36] using AWGS, and another [37] using EWGSOP criteria with AWGS cut offs. The study by Krenovsky et al. [34], included PRD participants while the remainder were exclusively in PD. The control groups were equally heterogeneous. Only one study [34] included age- and sex-matched controls. The study by Ozer et al. [35], included controls from a geriatrics outpatient department, Tan et al. 2018 and 2020 [36, 37] included spousal/sibling controls, and Yazar et al. [13] included hospital staff as a control population.

### The relationship between PD/PRD disease stage and prevalence of sarcopenia

**Fig. 3:**
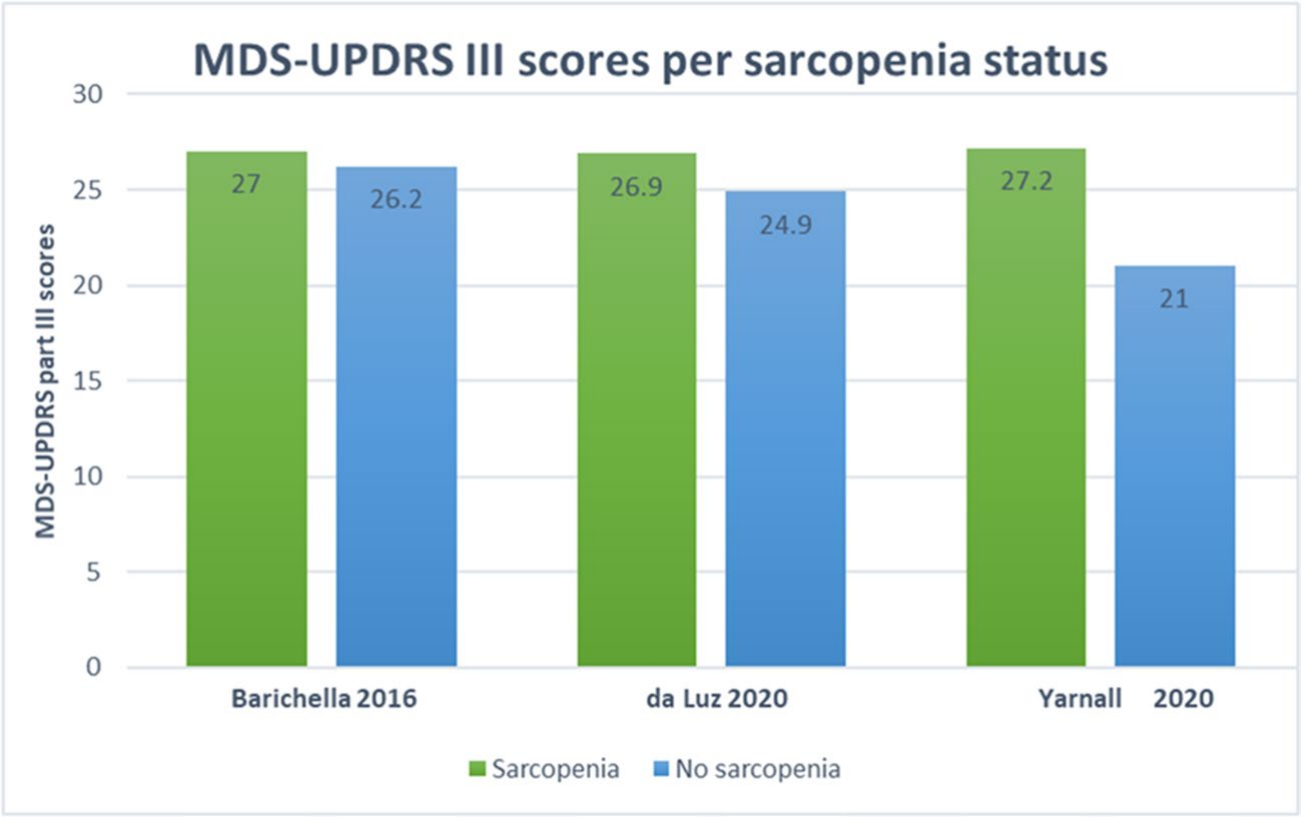
MDS-UPDRS part III scores by confirmed sarcopenia status. Values for MDS-UPDRS III for those with probable and confirmed sarcopenia reported. Mean scores reported

**Fig. 4:**
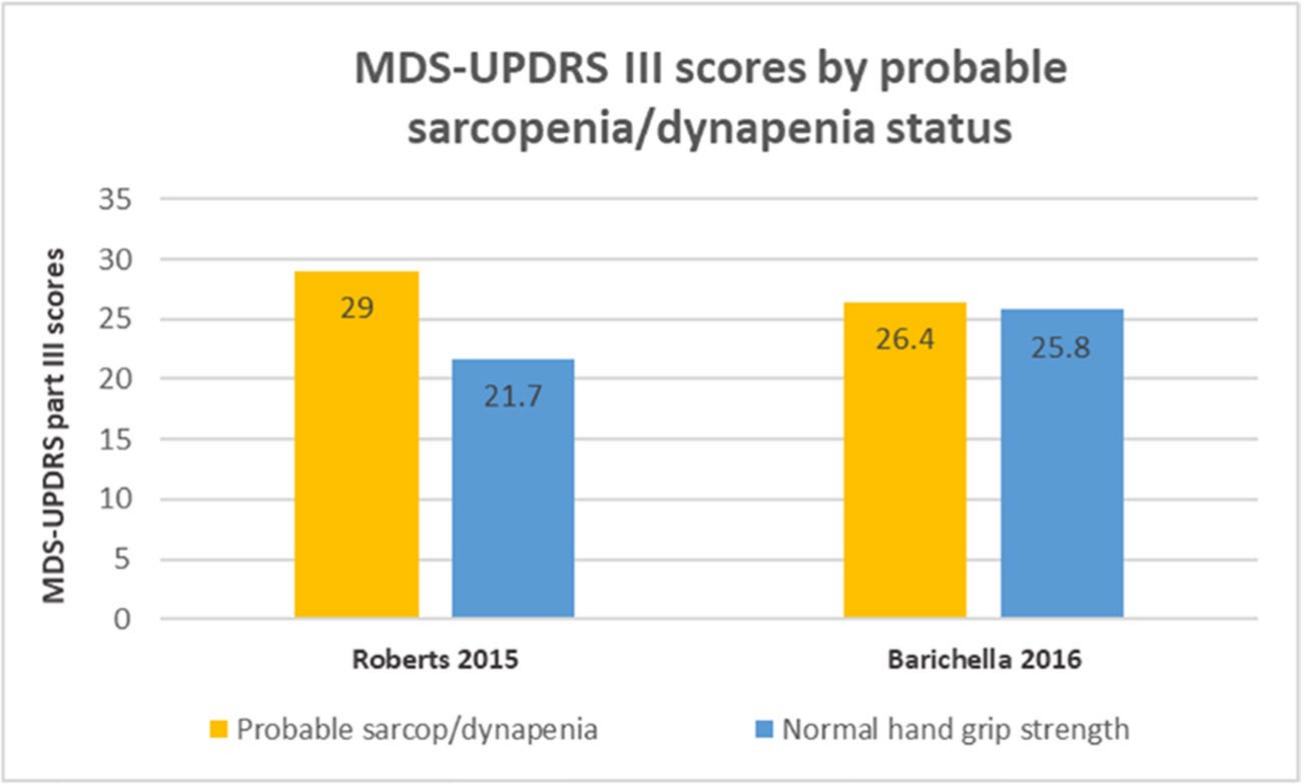
MDS-UPDRS III scores by probable sarcopenia or dynapenia status. Data from Roberts 2015 estimated using PlotDigitizer Software. Probable sarcopenia calculated using EWSOP2 cut-off. Barichella 2016 reported low hand grip strength (dynapenia) as per EWSOP cut-offs

Eight studies reported the mean MDS-UPDRS III scores for their population [12, 31–33, 36, 37, 39, 40]; two included PRD in their sample [31, 32]. From these eight studies, only three provided MDS-UPDRS III scores by confirmed sarcopenia status [12, 32, 33] and two by probable sarcopenia/low hand grip strength [32, 40]. The remaining three studies [31, 36, 37] provided MDS-UPDRS III scores for their whole parkinsonian population (i.e., not by sarcopenia status). Visually, it appeared that participants with confirmed sarcopenia had higher MDS-UPDRS III scores compared to those without sarcopenia (Fig. 3), although the difference was not clinically significant. Differences on MDS-UPDRS III by probable sarcopenia [40] (as per EWGSOP2 criteria) and low hand grip strength [32] status (as per EWGSOP criteria) are shown in Fig. 4. Data from Robert et al. [40] was estimated using the Plot Digitizer software. Due to the high degree of methodological heterogeneity further statistical testing was not performed.

Studies reporting on associations between sarcopenia (or components of sarcopenia) and MDS-UPDRS III scores presented contrasting conclusions. Barichella et al. 2016 [32] reported that participant with sarcopenia had longer disease duration, greater severity (Hoehn &Yahr stage) and higher disability as measured by the MDS-UPDRS II (motor experiences of activities of daily living; *p* < 0.05 for all comparisons). They found no association between hand grip strength or gait speed with motor severity, although participants with sarcopenia tended to have higher UPDRS III scores compared to non-sarcopenic participants (mean[sd] 27.0[10.7] and 26.2[10.0], respectively; *p* > 0.05). Similarly, da Luz et al. [33] found that participants with sarcopenia had higher MDS-UPDRS II (*p* < 0.05) but non-significantly greater MDS-UPDRS III scores, and that grip strength was inversely correlated with the MDS-UPDRS parts II and MDS-UPDRS II + III (r: -0.41 and -0.32, respectively; *p* < 0.05 both). However, a positive correlation was found between grip strength and MDS-UPDRS III (r: 0.229; *p* < 0.05). The authors also reported that in a multivariable regression model looking at predictors of SARC-F (a 5-item self-administered questionnaire to screen for sarcopenia risk [44]) that included variables such as age, grip strength, walk speed and UDPRS II and III scores, the UPDRS II score was the only variable to reach statistical significance. Further multivariable regressions models by Tan et al. 2018 [36] examining predictors of sarcopenia only found a statistically significant association with age, whilst variables such as gender, disease duration, MDS-UPDRS I, III and IV, PD motor phenotype and cognitive score were non-significant.

### Difference in prevalence between PD and PRD

Three studies presented data on sarcopenia in PRD. Barichella et al. [31] included participants with PD, PSP, MSA and vascular parkinsonism. However, no data were available on the number of participants in each group or the prevalence of sarcopenia in each individual group. The overall prevalence of sarcopenia was 2%.

In a separate study, the same authors [32] had a broader study population which included participants with PD (*n* = 265), vascular parkinsonism (*n* = 41), PSP (*n* = 38), MSA (*n* = 25), Lewy body disease (*n* = 13), CBD (*n* = 7) and participants defined as ‘other’(*n* = 5). The prevalence of sarcopenia in the PD cohort was 6% and in the ‘other parkinsonism’ group was 7.8% (*p* > 0.05). However, dynapenia (defined as handgrip strength < 30 kg in men and < 20 kg in women and consistent with cut off proposed by EWGSOP for low muscle strength) was more frequent in PRD (*p* < 0.01).

In the study by Krenovsky et al. [34], which included participants with PD (*n* = 53), dementia with Lewy bodies (*n* = 10), PSP (*n* = 2), CBS (*n* = 3), MSA (*n* = 6), and age- and sex-matched controls (*n* = 30), the prevalence of sarcopenia was almost four times higher in the PRD group than in the PD group (28.6% vs 7.5%, p = 0.017). This was consistent with findings of lower muscle strength in the PRD group (mean difference almost 5 kg lower). Interestingly, the authors also noted that the mean [SD] of skeletal muscle index (SMI) was higher in PD and PRD groups combined than that of the control group (9.3 [2.1] versus 8.5 [1.4] kg/m^2^, respectively; p-value not reported). This effect was mainly driven by PD participants (SMI mean [SD] for PD and PRD cohort 9.5 [2.1] and 8.8 [2.1] kg/m^2^, respectively). It is important to note however, that participants with PD had a considerably lower mean disease duration than the PRD group (61 vs 103 months) and had a higher number of muscle motor units (measured via electromyogram and by the motor unit number index [MUNIX]; p-values not reported), which may have positively impacted in the finding of higher muscle mass. In addition, despite the finding of lower SMI in the control group, they had better scores in muscle strength, gait distance, and in none of them sarcopenia was observed.

### How the prevalence of sarcopenia and probable sarcopenia in parkinsonian disorders compare with the general population

Supplementary Figures-1 and -2 show the prevalence of sarcopenia in the included studies according to EWGSOP and EWGSOP2 guidance. The horizontal line on each graph (i.e., 22% and 10% in Supplementary Figure-1 and -2, respectively) represents the estimates of sarcopenia prevalence in the general population according to Petermann-Rocha et al. systematic review and meta-analysis, which included 151 studies [29]. From the studies identified in our review which used the EWGSOP guidance, those including PD only participants all had an estimation of the prevalence of sarcopenia that was greater than the 22% prevalence estimate for the general population. The three studies that were below this line included PRD participants [31, 32, 34], although from the data available we cannot ascertain a negative relationship between PRD and sarcopenia. Using EWGSOP2 guidance, all studies with PD participants had an estimated sarcopenia prevalence that was greater than the 10% prevalence estimate for the general population.

Supplementary Figure-3 compares the prevalence of probable sarcopenia as per EWGSOP2 guidance in PD participants. The horizontal reference line represents the prevalence of probable sarcopenia in the general population (27%), estimated from a study of 3219 participants published in 2021 [30]. Three out of four studies in the PD populations had a greater prevalence of probable sarcopenia than the estimate for the general population.

Supplementary Figure-4 shows the prevalence of participants with PD and/or PRD who fall below the cut off for low grip strength according to EWGSOP. The same reference line of the estimate of probable sarcopenia by Trevisan et al. [30] is included. Five out of six studies in PD had a greater proportion of participants below cut off for low grip strength than studies in the general population. This is particularly noteworthy as the studies in the general population used the less conservative EWGSOP2 cut offs.

## Discussion

### Summary of main results

#### Highlights

This is the first review to systematically summarise the prevalence of sarcopenia in both PD and PRD using definitions proposed by internationally recognised sarcopenia working groups. It is also the first to assess how sarcopenia prevalence relates to PD disease severity. We found sarcopenia, and particularly probable sarcopenia (low skeletal muscle strength), to be a prevalent condition in PD and PRD. There was a trend suggesting that participants with sarcopenia tended to score higher in the MDS-UPDRS III. A similar trend could be observed for those with probable sarcopenia or reduced strength. However, in both cases these differences were small, and very few studies reported UPDRS-III scores by sarcopenia status which precluded the option of making more accurate assessments. We also found that sarcopenia was more common in parkinsonian disorders compared to controls.

#### Prevalence of sarcopenia

In the included studies, the prevalence of confirmed sarcopenia in participants with any parkinsonian disorder ranged from 2–31.4%. Including just studies with PD participants, the range was 10.9–31.4%. The two studies reporting the lowest prevalence of sarcopenia (≤ 6%) were from the same group and included a broader population of parkinsonian conditions [31, 32]. Of note, the RCT by Barichella et al., [31] tested the potential benefits of whey protein–based nutritional supplement and a physical programme in “nonsarcopenic patients”. Motor disease severity was mild, with a mean MDS-UPDRS III of 22.9. However, since the authors did not actively exclude participants with sarcopenia (2% of participants were sarcopenic), we decided to include these values.

A meta-analysis by Petermann-Rocha et al. found the prevalence of sarcopenia in the general population by EWGSOP, EWGSOP2 and AWGS classifications to be 22%, 10% and 15%, respectively [29]. The mean age across studies included in this meta-analysis was 71.5 years. An earlier systematic review and meta-analysis found that the overall prevalence of sarcopenia in community dwelling populations worldwide to be 10% [45]. Similarly, in 2021 Trevisan et al. reported that the prevalence of sarcopenia in a cohort of 3219 participants (96.4% community-dwelling) was 9.7% [30].

In our review, the five studies in which estimates of sarcopenia in PD population was compared to non-parkinsonian controls, all had odds ratios indicating a higher prevalence in parkinsonian participants (Fig. 2). This finding confirms our analyses where we contrast the prevalence of sarcopenia from several of the studies included in this review with estimates previously published from the general population (Supplementary Figs. 1–4).

#### Prevalence of probable sarcopenia

Among the sarcopenia working groups, the EWGSOP (i.e., pre-sarcopenia as per low muscle mass), EWGSOP2 (i.e., probable sarcopenia as per low muscle strength), and AWGS (i.e., possible sarcopenia as per low muscle mass or performance) have provided definition for cases in which parameters of sarcopenia are observed, but where not all diagnostic criteria are fulfilled for a definitive diagnosis. The importance of screening and early detection of sarcopenia has been emphasized by the EWGSOP2 guidelines [4], to aim at an early definitive diagnosis and subsequent intervention even at early stages. This is particularly important as sarcopenia is known to develop across the life-course and so opportunities for intervention exist at its earliest stages [46].

In our review, we found that the prevalence of probable sarcopenia, as defined by the EWGSOP2 criteria, ranged from 23.9–66.7% in the PD population (Supplementary Figure-3). The prevalence of low hand grip strength as per EWGSOP was 26.2–76.7%. Trevisan et al. [30] reported that the prevalence of probable sarcopenia to be 27% in a population with a mean age of 74.2 years. Similarly, a cross-sectional study of community dwelling Swiss older people found that 26.3% of females and 28% of males met the EWGSOP2 criteria for probable sarcopenia [47]. Despite the fact that the population in the latter study was considerable older (mean age of 84.9 [females] and 82.6 [males] years) than those included in our review (mean age 69.9 years) and that the risk of sarcopenia is inherent to ageing, in our review, we found estimates of probable sarcopenia that went as high as over twice times those found in the article by Wearing et al. [47] and Trevisan et al. [30]. This suggests a greater risk of muscle strength impairment in parkinsonian disorders compared to the general population.

In recent years there has been an increased understanding of the importance of muscle strength over mass when assessing sarcopenia outcomes [4]. This has also driven the focus on muscle strength in clinical research [3]. While there has been an inconsistent relationship demonstrated between low muscle mass and disability [48], the association between lower grip strength (a common indicator of overall muscle strength) and several physical, functional and cognitive health outcomes has been widely demonstrated [46]. It could also be argued, that including parameters reliant on walking speed to confirm possible sarcopenia (such as in the AWGS guideline) or sarcopenia severity may not be the most appropriate criterion to use in a parkinsonian population where slowness of movement (i.e., walking) and rigidity are fundamental features [2]. For instance, da Luz et al. [42] has found that both the SARC-F and SARC-CalF (questionnaires highly based on self-reported physical function) had low sensitivity (albeit high specificity) to identify sarcopenia in a PD cohort. These statements, in addition to the relatively lower cost-related equipment and training required to perform strength assessments, further support the use of muscle strength measurements as potentially a more suited modality to test for sarcopenia in PD and PRD.

#### Relationship to PD severity

There was a trend suggesting that participants with confirmed sarcopenia tended to score higher in the MDS-UPDRS III than participants without sarcopenia, although only three studies allowed this comparison [12, 32, 33] (Fig. 3). It is important to consider that, even though the MDS-UPDRS III [15] is a motor examination that assesses physical components such as upper and lower limb agility, physical function (e.g., arising from chair, gait, general body bradykinesia) and posture, it also includes the assessment of signs that may not be directly related to muscle strength, such as facial expression and speech. This may have impacted on the weak relationship found. In contrast, two authors [32, 33] reported statistically significant associations between the MDS-UPDRS II and sarcopenic status, posing the question whether the impairment of motor-related activities of daily living may be a better predictor of sarcopenia in this population. Interestingly, da Luz et al. [33], found a statistically significant positive association (worse outcomes) between the SARC-F questionnaire and MDS-UPDRS II scores, but not with MDS-UPDRS III scores. However, in this study PD participants with sarcopenia tended to have worse (higher) scores in sections II, III and II + III (means difference + 2.7 *p* < 0.05; + 2 *p* > 0.05; + 4.8 *p* > 0.05, respectively) of this test compared to non-sarcopenic PD participants, and the lack of statistical significance may have been associated to the considerably lower number of sarcopenic PD participants (*n* = 15) compared to non-sarcopenic PD counterparts (*n* = 62). Barichella et al. [32], also reported a non-significant trend towards participants with sarcopenia scoring worse in the MDS-UPDRS III than non-sarcopenic participants. They also found that the ability to carry out a gait speed assessment was associated with parameters of sarcopenia (i.e., SMM and grip strength), and that among those with sarcopenia, 4.9% were able to perform this walk test compared to 11.2% of participants who were not. Similarly, in the study by Yarnall et al. [12], PD participants without sarcopenia, with probable and confirmed sarcopenia had an average MDS-UPDRS III score of 24.5, 26 and 29.5 (higher scores meaning higher PD-related impairments), respectively (p-values not reported). When considering all the components of the MDS-UPDRS (e.g., total test score), Vetrano et al. [38] found that in multivariable regression analysis severe sarcopenia was independently associated with having an above median total MDS-UPDRS score of > 42 (OR 2.30; 95% CI 1.15–4.58).

Though further research is needed before conclusions can be drawn, this early evidence suggests a possible relationship between PD disease stage and sarcopenia, and highlights the need for early case finding.

#### Prevalence in PD and PRD

There was insufficient evidence to draw conclusions about the prevalence of sarcopenia in PRD as compared to PD. However, there was a trend suggesting that sarcopenia (or components of sarcopenia) may be more prevalent in PRD populations. Barichella et al. [32] reported that the percentage of people with sarcopenia was a slightly higher in the PRD group (1.8% higher, *p* > 0.05). They also found that dynapenia (low muscle strength as per EWGSOP cut-off) was more prevalent in PRD (*p* < 0.01). They reported that their PD cohort was younger than the PRD group and had longer disease duration. The study by Krenovsky et al. [34], reported that the prevalence of sarcopenia in their PRD cohort was almost four times higher than in their PD group (28.6% vs 7.5%, *p* < 0.05). Grip strength was also considerably lower in the PRD group (26.7 vs 31.4 kg, p-value not reported). However, it is important to consider that in this study, participants with PD had a considerably lower mean disease duration than the PRD group (61 vs 103 months) which may have impacted in these results.

Future research focusing on the differences of sarcopenia status in these populations could help to delineate shared pathophysiology between sarcopenia and parkinsonian disorders.

### Comparison of findings

To our knowledge, only one systematic review and meta-analysis exists that addresses the question of the prevalence of sarcopenia in PD [24]. Cai et al. included 10 studies with PD participants. No PRD participants were included in this study and there was no assessment of the prevalence of probable sarcopenia. Several studies which were included in the review by Cai et al. were excluded in this review as sarcopenia was not defined by guidance from recognised working groups. The authors performed a random effects meta-analysis and found the prevalence of sarcopenia in Parkinson’s disease to be 6 to 55.5% with a pooled prevalence of 29%. When only studies at low risk of bias were considered, this dropped to 17%.

In this systematic review, we did not perform a meta-analysis due to the high degree of clinical, methodological and statistical heterogeneity across studies.

### Clinical implications

In this systematic review we demonstrate that sarcopenia is prevalent in parkinsonian disorders. Muscle weakness as measured by grip strength was more prevalent in parkinsonian participants than in controls, and their prevalence values were generally higher than estimates previously reported from the general population.

Recognition of a greater prevalence of sarcopenia in PD should prompt early clinical testing and provision of early interventions with a focus on prevention. This is especially important to the quality of life of those with a movement disorder to avoid the risk of negative clinical outcomes being compounded by a syndrome of muscular weakness.

These findings have important clinical relevance highlighting the importance of screening for sarcopenia in people with parkinsonian disorders. Increasing consensus is that sarcopenia should be managed using resistance exercises [49], although the evidence for treatment specifically in Parkinson’s disease is yet to be established.

This further highlights the need for early identification in people with Parkinson’s disease, at a time when addressing the components of pre-/probable sarcopenia (i.e., low muscle strength or muscle mass) could have several health benefits. The detrimental effects of sarcopenia have been well documented and include an increased risk of falling and fractures, impairment of independence and quality of life, increase of the likelihood of mobility disorders, for the need for long-term care placement, and death [4].

Further research is needed to strengthen these conclusions as a large degree of clinical and methodological heterogeneity was found across the included studies. A standardisation of definitions with appropriate cut offs to the population in question would aid comparison between studies [50]. The benefit of resistance exercise programs in sarcopenia has been demonstrated [5] but specific study of the benefit in PD and PRD populations is lacking.

### Limitations

We excluded studies whose populations were made exclusively of nursing home residents to avoid the presence of sarcopenia being confounded by frailty. More information is needed to characterise later stages of the disease and institutionalised individuals. Also, studies included in this review mostly focussed on participants who are not cognitively impaired. A systematic review by Waite et al. demonstrated an increase in sarcopenia in dementia [51], therefore excluding those with cognitive impairment may have led to an underestimate of sarcopenia. Lastly, we could not secure confirmation whether two of the studies included in this review [36, 37] were carried out on the same cohort of participants. However, considering that the studies differ in 20 PD participants we decided against exclusion.

A key limitation of sarcopenia research is that globally agreed upon guidelines for diagnosis although in preparation [50], are not yet available. This contributed to some of the methodological heterogeneity found in this review. While standardisation of guidance is needed, cut offs for individual parameters (e.g., muscle strength) need to be appropriate for the population under examination such as in Parkinson’s disease. This highlights the importance of reporting relevant parameters, according to those proposed by stablished sarcopenia working groups to define this syndrome.

Finally, we identified a high degree of clinical heterogeneity in control populations across studies. Hence, studies using better matched population are needed to make more reliable comparisons. Also, all the evidence included in this review is cross-sectional so causality cannot be implied. Future longitudinal research is needed to delineate the relationship between sarcopenia over the course of PD and related disorders.

### Quality of evidence

The median score was 15/20 (range 11–18) using the AXIS appraisal tool.

## Conclusions

This systematic review of cross-sectional studies in community dwelling adults has shown that the prevalence of probable sarcopenia, low muscle strength and confirmed sarcopenia is common in PD and PRD. Compared to people without parkinsonian disorders, studies using control groups consistently found higher prevalence of sarcopenia in their PD or PRD population; and comparisons against estimates in the general population show a similar trend. The associations between the presence of sarcopenia and its relationship to PD motor severity should be investigated further.

Early screening (through strength measurement), detection and treatment of probable and confirmed sarcopenia may potentially act as a protective measure for the reduction of strength and physical function that is likely occur in people with PD as the condition progress.

### Supplementary Information

Below is the link to the electronic supplementary material.

Supplementary file1 (DOCX 193 KB)
