# Supplementary material for: The prevalence of sarcopenia in Parkinson’s disease and related disorders- a systematic review
Source: Neurol Sci. 2023 Aug 18;44(12):4205–17. doi: 10.1007/s10072-023-07007-0 (PMC10641055; doi:10.1007/s10072-023-07007-0)
Supplement: Supplementary file 1 — Supplementary file1 (DOCX 193 KB) [file 10072_2023_7007_MOESM1_ESM.docx]

**Supplementary Materials**

Supplementary Table 1: Parameters and cut offs for defining sarcopenia as per EWGSOP, EWGSOP2, AWGS and IWGS

|  | | **EWGSOP [8]** |  | **EWGSOP2 [4]** |  | **AWGS [9]** |  | **IWGS^ [10]** |  |
| --- | --- | --- | --- | --- | --- | --- | --- | --- | --- |
| Purpose | | Tool | Cut off | Tool | Cut off | Tool | Cut off | Tool | Cut off |
| Case  finding | | Gait speed | ≤ 0.8 m/s | SARC-F questionnaire | ≥4 | SARC-F | ≥4 | - | - |
|  | |  |  | Ishii screening tool |  | SARC-CalF | ≥11 | - | - |
|  | |  |  | Patient report |  | Calf  circumference | M <34cm W <33cm | - | - |
| Measuring strength | | Grip strength | M<30kg  W<20kg | Grip strength | M <27kg W<16kg | Grip strength | M <28kg W<18kg | - | - |
|  | | knee flexion/ extension |  |  |  |  |  | - | - |
|  | | Peak expiratory  flow* |  | Chair stand test | >15s for  5 rises |  |  | - | - |
| Measuring  mass/quality | | DXA | 2SD below mean for healthy young adults | ASMM by DXA | M<20kg  W <15kg  or ASM/height: M<7kg/m^2^ W<5.5kg/m^2^ | Height adjusted muscle  mass DXA | M <7kg/m^2^ W<5.4kg/m^2^ | Appendicular fat lean mass/ height^2^ (aLM/Ht^2^) by DXA | M: 7.2 kg/ m^2^  W: 5.6 kg/ m^2^ |
|  | | BIA | as above | SMM or ASMM predicted by BIA | as above | Height adjusted muscle  mass BIA | M< 7kg/m^2^ W<5.7kg/m^2^ | - | - |
|  | | CT or MRI |  | SMM or ASMM by CT or MRI | as above |  |  | - | - |
|  | | Total or partial  body K+ per fat free soft tissue |  | Calf circumference** | <31cm |  |  | - | - |
|  | **Continuation** |  |  |  |  |  |  |  | |
|  | **EWGSOP [8]** |  | **EWGSOP2  [4]** |  | **AWGS [9]** |  | **IWGS^ [10]** |  | |
| Purpose | Tool | Cut off | Tool | Cut off | Tool | Cut off | Tool | Cut off | |
| Measuring | | TUG |  | TUG | ≥ 20s | Chair stand test | ≥12 s for 5  rises | 4mGS | <1m/s |
| physical  performance | | 6mGS | <1m/s |  |  | 6mGS | <1m/s | - | - |
|  | | 4mGS | <0.8m/s | 4mGS | <0.8m/s |  |  | - | - |
|  | | Stair climb  power test |  | 400 m walk test | Non completion  or ≥6 min |  |  | - | - |
|  | | SPPB | ≤8 points | SPPB | ≤8 points | SPPB | ≤9 | - | - |

Abbreviations: 4mGS 4-meter gait speed; 6mGS 6-meter gair speed; ASMM appendicular skeletal muscle mass; BIA bioelectrical impedance analysis; DXA dual energy X ray absorptiometry; SARC-F Strength, Assistance with walking, Rise from a chair, Climb stairs and Falls; SMM whole body skeletal muscle mass; SPPB short physical performance battery; TUG timed up and go; M men; W women

*Cannot be recommended as an isolated measure

**if no other muscle mass diagnostic methods available

EWGSOP2 also list the following as alternative or new tools:

- Mid-thigh, or lumbar muscle cross-sectional area by CT, ultrasound (US) or MRI
- Muscle quality by mid-thigh or total body muscle quality by muscle biopsy, CT, US, MRI or Magnetic resonance Spectroscopy (MRS)
- Creatine dilution test
- Isometric torque methods for lower limb when measurement of grip is not possible due to hand disability (e.g. with advanced arthritis or stroke

^The IWGS [10] recommend testing for sarcopenia in older patients with observed declines in physical function, strength or health status. Difficulties performing ADLs, a history of recurrent falls, recent weight loss, recent hospitalisation or those with chronic conditions associated with muscle loss. Especially those who are bedridden, non-ambulatory or who cannot independently rise from a chair. Anyone with a 4mGS <1m/s should be referred for DXA and those <20th percentile of values for healthy young adults are classified as sarcopenic or those with appendicular fat lean mass of M ≤ 7.23 kg/ m2 F ≤ 5.67.

Supplementary Table 2: Scores from critical appraisal tool to assess the quality of cross-sectional studies (AXIS).

| **Author (ref)** | **Study type** | **Total /20** |
| --- | --- | --- |
| Barichella [31] | RCT | 16 |
| Barichella [32] | CS | 16 |
| da Luz [33] | CS | 11 |
| Krenovsky [34] | CS | 12 |
| Ozer [35] | CS | 15 |
| Tan [36] | CS | 16 |
| Tan [37] | CS | 16 |
| Vetrano [38] | CS | 13 |
| Yarnall [12] | CS | 18 |
| Yazar [13] | CS | 11 |
| Bernhard [41] | CS | 15 |
| Lindskov [39] | Cohort | 11 |
| Roberts [40] | CS | 18 |
| Pessoa Lima [17] | CS | 15 |

CS= cross-sectional study, RCT= randomised control trial, ref= reference

Supplementary Figure 1: Prevalence of sarcopenia in PD and PRD participants (%) compared to the general population using EWGSOP criteria


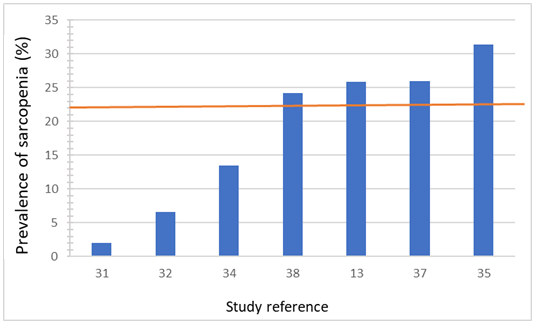


The prevalence of sarcopenia in PD and PRD populations using EWGSOP guidance with reference line showing estimated prevalence of sarcopenia in the general population (i.e., 22%) using EWGSOP guidance as per Petermann-Rocha et al [29]. Studies marked with an * include PRD in their population sample. Studies referenced: 2019 Barichella et al., [31], 2016 Barichella et al., [32], Krenovsky et al., [34], Vetrano et al., [38], Yazar et al., [13], 2020 Tan et al., [37], Ozer et al., [35],

Supplementary Figure 2: Prevalence of sarcopenia (%) in PD and PRD participants compared to the general population using EWGSOP2 criteria


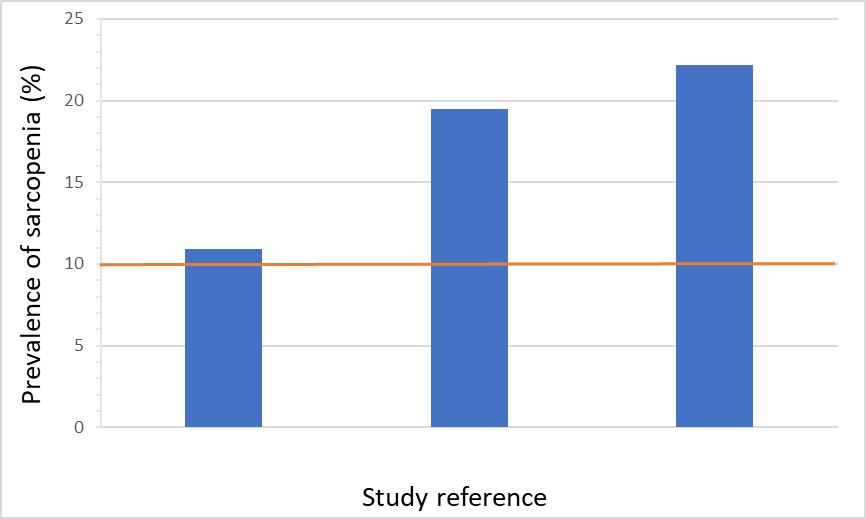


41 33 12

Study Reference

The prevalence of sarcopenia in PD populations using EWGSOP2 guidance with reference line showing estimated prevalence of sarcopenia in the general population (i.e., 10%) using EWGSOP2 guidance as per Petermann-Rocha et al [29]. Studies referenced: Bernhard et al., [41]; da Luz et al., [33]Yarnall et al., [12]

Supplementary Figure 3: Prevalence of probable sarcopenia (%) of PD participants compared to the general population using EWGSOP2 criteria


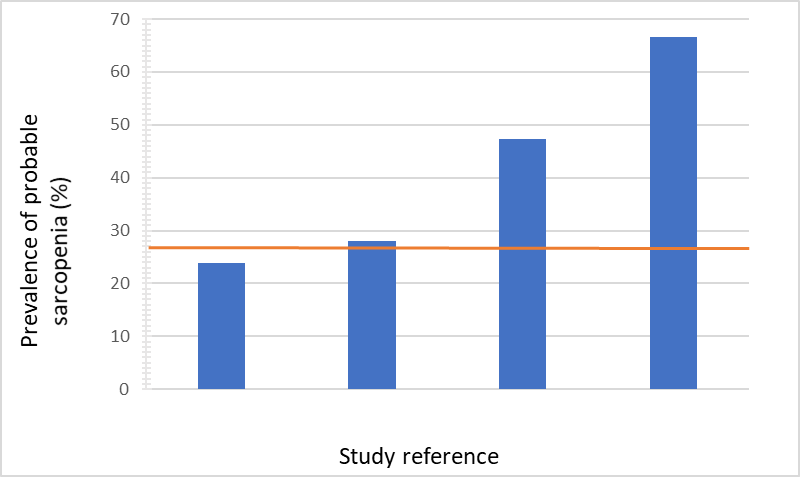


41 40 17 12

The prevalence of probable sarcopenia in % PD populations using EWGSOP2 guidance with reference line showing estimated prevalence of probable sarcopenia in the general population (i.e., 27%) using EWGSOP2 guidance as per Trevisan et al., [30]. Studies referenced: Bernhard et al., [41]; Roberts [40]; Lima et al., [17]; Yarnall et al., [12]

Supplementary Figure 4: Prevalence of probable sarcopenia in PD and PRD participants (% of participants) using EWGSOP2 criteria with EWGSOP cut offs compared to the general population using EWGSOP2 criteria


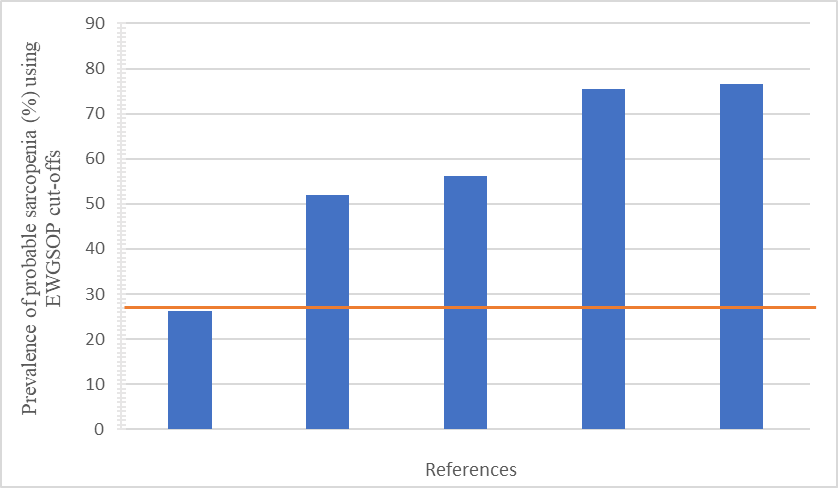


39 31* 37 32* 38

The prevalence of probable sarcopenia in PD and PRD populations using EWGSOP cut offs with reference line showing estimated prevalence of probable sarcopenia in the general population (i.e., 27%) using EWGSOP2 guidance as per Trevisan et al., [30]. Studies marked with an * include PRD in their population sample. Studies referenced: Lindskov et al., [39]; Barichella et al., [31]; Tan et al., [37]; Barichella et al., [32], Vetrano et al., [38]
